# The Editor's Letter-Box

**Published:** 1919-01-04

**Authors:** Geraldine Bremner

**Affiliations:** Nurse Representative on the Management, Nurses' Co-operation, and a member of the College of Nursing.


*To the Editor of* THE HOSPITAL.

SIR,—In answer to "Ierne's" letter in THE HOSPITAL of December 28, I would first say that I am a member of a very large body of nurses in London, and therefore have ample opportunity of hearing and discussing nursing subjects with my fellow-workers; and from what I hear I most emphatically say that "Ierne" is voicing the opinion of the rank and file of great numbers of the nursing profession. It is very evident that there is a growing distrust in the College of Nursing, not only amongst those outside its portals, but also amongst many of its members. There is a very strong feeling that through it no reconstruction is taking place in the (present) nursing world, and that the Executive of the College are working for and spending the funds of the College only for the interest and good of the future nurse, with little or no thought for those who have borne and are bearing the heat and burden of the day.

All right-minded members of the profession approve of a scheme in the College curriculum to help future members of the profession, but they did not subscribe £10,000 for that object to be first and foremost, nor did the public give its generous support for that object alone. Was it not the nation's tribute fund for nurses (*for services rendered*)? May the College, before it is too late, "wake up" and initiate reconstruction in the nursing world, and first and foremost make the lives of the present members of the profession more bearable, and so swell its ranks. If this is not done its members will soon disown it and look for reconstruction in other directions.

GERALDINE BREMNER,
Nurse Representative on the Management, Nurses' Co-operation, and a member of the College of Nursing.

